# Determinants of metabolic dysfunction associated steatotic liver disease and liver fibrosis in patients with type 2 diabetes mellitus: a cross-sectional study

**DOI:** 10.3389/fmed.2026.1863576

**Published:** 2026-07-17

**Authors:** Soumaya Mrabet, Ghada Saad, Asma Ben cheikh, Mohamed Abu Awad, Imen Halloul, Raida Harbi, Meryam Eloillaf, Imen Akkari, Yosra Hasni, Elhem Ben Jazia

**Affiliations:** 1 Department of Gastroenterology Farhat Hached University Hospital, Faculty of Medicine, University of Sousse, Sousse, Tunisia; 2 Department of Endocrinology Farhat Hached University hospital, Faculty of Medicine, University of Sousse, Sousse, Tunisia; 3 Department of Preventive and Community Medicine, Sahloul University Hospital, Faculty of Medicine of Sousse, University of Sousse, Sousse, Tunisia

**Keywords:** fibroscan, hepatic steatosis, liver fibrosis, MASH, MASLD, type 2 diabetes mellitus

## Abstract

**Background:**

Metabolic dysfunction-associated steatotic liver disease (MASLD), formerly called non-alcoholic fatty liver disease (NAFLD), is today the leading cause of chronic liver conditions in the context of a global epidemic of obesity and metabolic disorders. Our study aims to determine the prevalence of MASLD and advanced liver fibrosis as well as the associated factors in patients with type 2 diabetes Mellitus (T2D).

**Methods:**

Cross-sectional study including 149 patients with T2D, conducted in the Endocrinology-Diabetology and Hepato-Gastroenterology departments over a period of 6 months. Screening for steatosis and liver fibrosis was carried out using FibroScan.

**Results:**

The average age of the patients was 57.9 ± 11.8 years, with a female predominance (63.8%). Based on the EASL cutoffs, the MASLD ratio was 69.1% [95% CI (61.7–76.5], *n* = 103). Multivariable analysis identified high Body Mass Index [aOR = 1.25; 95% CI (1.13–1.38); *p* < 0.001] and total cholesterol [aOR = 1.69; 95% CI (1.15–2.48); *p* = 0.007] as independent predictors of MASLD. The median liver stiffness measurement was 5.0 kPa (IQR 4.0–6.5). Advanced fibrosis was confidently ruled out (< 8 kPa) in 82.6% (*n* = 123) of patients. Advanced fibrosis could not be ruled out (≥8 kPa) in 17.4% [95% CI (11.7-24.5), *n* = 26], which included 13.4% (*n* = 20) in the intermediate-risk zone (8–11.99 kPa) and 4% (*n* = 6) at high risk of advanced fibrosis (≥12 kPa). Higher platelet distribution width (PDW) was associated with nearly a threefold increase in the odds of advanced fibrosis (aOR = 2.78; 95% CI: 1.08–7.21; *p* = 0.035), while increasing GGT levels were also significantly associated with advanced fibrosis (aOR = 1.12; 95% CI: 1.03–1.21; *p* = 0.006).

**Conclusion:**

In our study, we found that 69.1% of participants met the criteria for MASLD, and advanced fibrosis could not be ruled out in 17.4% of patients, with 4% being at high risk (≥12 kPa). It is essential to identify these at-risk patients who require multidisciplinary management in order to prevent progression to cirrhosis and its complications.

## Background

Metabolic dysfunction associated steatotic liver disease (MASLD), formerly known as non-alcoholic fatty liver disease (NAFLD), is currently the most common chronic liver disease worldwide. According to the recent multisociety Delphi consensus, the term MASLD has replaced NAFLD to better reflect the underlying metabolic dysfunction associated with hepatic steatosis. MASLD is defined by the presence of hepatic steatosis in at least 5% of hepatocytes, together with at least one cardiometabolic risk factor and in the absence of other specific causes of liver disease. The disease spectrum ranges from simple steatosis to metabolic dysfunction-associated steatohepatitis (MASH), advanced fibrosis, cirrhosis, and hepatocellular carcinoma (1, 2). The global prevalence of MASLD is around 25% (3). This prevalence continues to rise with the growing epidemic of obesity and metabolic syndrome, particularly type 2 diabetes (T2D), with a prevalence reaching 60–75% (4).

The main condition for the genesis of T2D is insulin resistance, which is an important factor in the progression of MASLD, thus explaining the close link between MASLD and T2D (5). Several studies have demonstrated a bidirectional relationship between MASLD and T2D. On the one hand, MASLD increases the risk of developing T2D and the risk of macrovascular and microvascular complications in patients with T2D (6, 7). On the other hand, diabetic patients tend to progress more rapidly to advanced forms, including non-alcoholic steatohepatitis (NASH), recently named metabolic-associated steatohepatitis (MASH), advanced liver fibrosis, cirrhosis, and hepatocellular carcinoma (HCC) (8).

The degree of liver fibrosis is the most important predictor of the progression of MASLD, with significant liver fibrosis being defined by a fibrosis score greater than or equal to F2, thus defining at-risk MASH patients (9). The majority of guidelines, particularly the EASL (European Association for the Study of the Liver), the AASLD (American Association for the Study of Liver Diseases), and the ADA (American Diabetes Association), consider patients with T2D to be at high risk of developing MASLD and recommend screening liver disease in these patients (2, 10, 11). The screening guidelines would likely be difficult or poorly implemented due to the unavailability of all screening methods at the various centers.

Given the risk of progression to MASH, liver fibrosis, cirrhosis, and HCC, it is crucial to identify and select patients at high risk of developing liver complications in order to manage MASLD and prevent these complications.

The aim of our study was to determine the prevalence and factors associated with MASLD and liver fibrosis in patients with T2D.

## Methods

### Study design and sample

A prospective descriptive cross-sectional study was conducted in Hepato-Gastroenterology and Endocrinology-Diabetology departments. Enrollment took place from January 2024 to June 2024. Eligible participants were adults aged ≥18 years with type 2 diabetes mellitus, recruited from the Endocrinology–Diabetology department.

Patients were not included if they had hepatitis B or C, non-metabolic chronic liver diseases (primary biliary cholangitis, primary sclerosing cholangitis, autoimmune hepatitis, or overlap syndromes), hepatocellular carcinoma or other hepatic malignancies, Budd–Chiari syndrome or portal vein thrombosis, excessive alcohol consumption (>30 g/day in men and >20 g/day in women), exposure to steatogenic drugs or medications known to induce liver fibrosis, or a history of illicit drug use. In addition, patients lacking FibroScan were also excluded.

Among the initial sample of patients with T2D assessed eligibility (*n* = 184), 35 patients were excluded. Exclusion criteria comprised: excessive alcohol consumption (*n* = 1), hepatitis B virus infection (*n* = 2), primary biliary cholangitis (*n* = 2), refusal to undergo abdominal ultrasound (*n* = 5), refusal to undergo FibroScan (*n* = 9), and loss to follow-up before completion of the required investigations (*n* = 16). Consequently, 149 patients (81.0% of the screened diabetic population) were included in the final analysis. All included patients underwent Fibroscan examination; no-preliminary no-invasive tests (FIB-4 score or liver enzyme measurement) were used to select candidates for transient elastography.

The sample size was calculated based on the expected prevalence of the primary outcome, MASLD, among individuals with T2D, estimated at 89.7% in previous studies (12).

Assuming a 95% confidence level (*z* = 1.96) and a margin of error of 5%, the minimum required sample size was calculated to be 142 participants. To account for a 5% non-response rate, the final sample size was increased to 149 participants. All participants provided written informed consent.

### Data collection

Data was collected using a standardized case report form. Clinical variables included age, sex, cardiovascular comorbidities (hypertension, stroke, ischemic heart disease, peripheral arterial disease, and dyslipidemia), anthropometric measurements (weight, height, body mass index (BMI), and waist circumference, blood pressure, antidiabetic treatment, and chronic diabetic macrovascular and microvascular complications (peripheral arterial disease, stroke, coronary artery disease, diabetic nephropathy, neuropathy, and retinopathy).

Laboratory assessments comprised liver function tests (AST, ALT, ALP, gamma-glutamyl transferase (GGT), total bilirubin), prothrombin time, serum albumin, lipid profile, complete blood count, fasting plasma glucose, and HbA1c.

All patients underwent transient elastography (FibroScan^®^, Echosens) at the Gastroenterology Department of Farhat Hached University Hospital to assess hepatic steatosis using the controlled attenuation parameter (CAP) and liver fibrosis using liver stiffness measurement (LSM). M or XL probes were used, the latter for obese patients (BMI ≥30 kg/m^2^) or when the skin-to-liver capsule distance exceeded 25 mm. Measurements were considered reliable with ≥10 valid acquisitions, interquartile range < 30%, and success rate >60%.

### Operational definitions of variables

T2DM was diagnosed according to the ADA criteria (13), based on fasting plasma glucose ≥126 mg/dL, 2-h plasma glucose ≥200 mg/dL after a 75 g oral glucose tolerance test, HbA1c ≥6.5%, or classic hyperglycemia symptoms with a random plasma glucose ≥200 mg/d.

MASLD was diagnosed in patients with hepatic steatosis and at least one metabolic risk factor, after excluding secondary causes. Steatosis was assessed using controlled attenuation parameter (CAP). According to the EASL cutoffs, a CAP value ≥248 dB/m was used to define the presence of hepatic steatosis (S1). Steatosis was further graded as S1 (248–267 dB/m), S2 (268–279 dB/m), and S3 (≥280 dB/m) (10).

Liver fibrosis was assessed by FibroScan^®^ LSM. Dual cutoffs (8 and 12 kPa) were applied for fibrosis stratification. A LSM < 8 Kpa defines the cutoff to rule out advanced fibrosis. A LSM ≥8 kPa was used to define patients in whom advanced fibrosis could not be excluded, while an LSM ≥12 kPa identified patients at high risk of advanced fibrosis (10).

Hypertension was defined according to ESC/ESH 2018 guidelines as systolic blood pressure ≥140 mmHg and/or diastolic blood pressure ≥90 mmHg, based on at least two separate measurements taken on two different occasions, or current use of antihypertensive medication. Patients taking antihypertensive drugs were considered hypertensive regardless of their measured blood pressure values. (14) Dyslipidemia was defined by a history of dyslipidemia or abnormal lipid profile, including LDL ≥130 mg/dL, HDL < 40 mg/dL, total cholesterol >200 mg/dL, or triglycerides ≥1.7 mmol/L. (15) Obesity was defined by BMI ≥30 kg/m^2^ and classified as class I (30–34.9), class II (35–39.9), or class III (≥40); abdominal obesity was defined as waist circumference ≥94 cm in men and ≥80 cm in women (16).

### Statistical analysis

In the descriptive analysis, categorical variables were described using absolute frequencies with percentages and 95% confidence intervals (95% CI) Quantitative variables were expressed as means and standard deviations (SD) when normally distributed; otherwise, they were presented as medians and interquartile ranges (IQR). Comparisons were performed using chi-square or Fisher's exact tests for categorical variables and Student's *t*-test or Mann–Whitney *U*-test for continuous variables. Variables with *p* < 0.2 in univariate analysis were included in a binary logistic regression model using the stepwise Hosmer–Lemeshow method to identify independent risk factors for steatosis and MASLD at risk of progressive disease.

The absence of multicollinearity was verified using Jamovi software (version 2.7.31), with all VIF values below the acceptable threshold of 5. A *p*-value < 0.05 was considered statistically significant. Adjusted odds ratios (aORs) were therefore used to estimate the independent effect of each factor on MASLD and significant fibrosis.Receiver operating characteristic (ROC) curve analysis, using the Youden index, was performed to identify optimal cut-off values for quantitative variables associated with MASLD or severe fibrosis. Analyses were performed using SPSS version 17.0.

## Results

### Baseline characteristics

Among 149 patients with type 2 diabetes mellitus, the mean age was 57.9 ± 11.8 years, and 63.8% were women (sex ratio 0.56). The mean body mass index was 30.6 ± 5.4 with obesity observed in 51.4% of participants and abdominal obesity in 82.5%. Overall, 78.5% of patients had at least one comorbidity, with arterial hypertension being the most frequent (59.1%). Oral antidiabetic therapy alone was used in 52.3% of participants, insulin therapy in 9.4%, and a combination of oral agents and insulin in 33.6%. Chronic diabetic complications were observed in 38.9% of patients. Biological parameters are summarized in Table 1. Median fasting plasma glucose was 10.4 mmol/L (IQR 8.2–13.9), and median HbA1c was 9.4% (8.0–10.8). Table 1 summarizes the demographic, clinical, biological, and elastographic characteristics of the study population.

**Table 1:** Demographic, clinical and biological characteristics of study participants.

Variables	*N*	%
**Age, mean ±SD**	57.9 ± 11.8	
Sex		
Female	95	63.8
Male	54	36.2
Lifestyle habits		
Smoking	25	16.8
Alcohol consumption	01	00.7
Antidiabetic treatment		
Oral antidiabetic drugs	78	52.3
Insulin	14	09.4
Oral antidiabetic drugs and insulin	50	33.6
Diet alone	07	04.7
**Insulin**	64	42.9
**Degenerative complications**	**58**	**38.9**
• Retinopathy	32	55.2
• Nephropathy	21	36.2
• Neuropathy	34	58.6
• Stroke	04	07.4
• Peripheral arterial disease of the lower limbs	07	12.9
• Acute coronary syndrome	13	22.5
**BMI (kg/m**^**2**^**), mean ±SD** (*n* = 144)	30.6 ± 5.4	
• Normal weight		
• Overweight	19	13.2
• Obesity	51	35.4
**Obesity**	74	51.4
• Class I	45	60.8
• Class II	22	29.7
• Class III	07	09.5
**Abdominal obesity** (*n* = 143)	118	82.5
Waist circumference (cm), median [IQR]		
Female	100.0[95.0–108.0]	
Male	98.0[89.2–105.0]	
**Systolic blood pressure (SBP) (mmHg), median [IQR]**		130.0[120.0–140.0]
**Diastolic blood pressure (DBP) (mmHg), median [IQR]**		70.0[70.0–80.0]
**White blood cell count/mm**^**3**^ **(WBC), median [IQR]**		7200.0[5500.0–8500.0]
**Hemoglobin (g/dL) (Hb), median [IQR]**		13.0[11.9–13.9]
**Platelet count/μL (PLT), median [IQR]**		238000[166000–278000]
**Platelet distribution width (PDW) (%), median [IQR]**		9.4[8.9–10.0]
**Fasting blood glucose (mmol/L), median [IQR]**		10.4[8.2–13.9]
**Glycosylated hemoglobin (HbA1c)(%), median [IQR]**		9.4[8.0–10.8]
**Triglycerides (mmol/L), median [IQR]**		1.4[1.1–1.9]
**Total cholesterol (mmol/L), median [IQR]**		4.3[3.6–5.3]
**High density lipoprotein (HDL) cholesterol (mmol/L), median [IQR]**		1.1[1–1.3]
**Aspartate aminotransferase (AST) (UI/L), median [IQR]**		20.0[15.0–25.0]
**Alanin aminotransferase (ALT) (UI/L), median [IQR]**		17.0[12.2–26.0]
**Gamma–glutamyl transferase (GGT) (UI/L), median [IQR]**		23.0[17.0–34.0]
**Alkaline phosphatase (ALP) (UI/L), median [IQR]**		74.5[60.2–91.0]
**Total bilirubin (UI/L), median [IQR]**		10.0 [7.0–12.0]
**Prothrombin time(%), median [IQR]**		99.0[93.0–100.0]
**Albumin (g/L), median [IQR]**		42.0[40.0–45.0]
**Ferritin (μg/ L), median [IQR]**		56.0[31.5–80.2]

### Prevalence of MASLD and risk factors

The mean CAP value was 271.3 ± 55.3. Using the EASL-recommendation threshold of CAP ≥ 248 dB/m, the MASLD ratio was 69.1% [95% CI (61.7–76.5), *n* = 103]. Steatosis grading showed that 44.3% of patients (*n* = 66) had grade 3 steatosis (S3), 12.1% (*n* = 18) had grade 2 steatosis (S2), 12.8% (*n* = 19) had grade 1 steatosis (S1), while 30.9% (*n* = 46) had no steatosis (S0).

In univariate analysis, MASLD was associated with younger age (*p* = 0.019), higher BMI (*p* < 0.001), obesity (*p* < 0.001) and a higher waist circumference (*p* < 0.001). Patients with MASLD had higher triglyceride and total cholesterol levels (both *p* = 0.001), and higher ALT (*p* = 0·018) and GGT levels (*p* = 0·006). CAP values and liver stiffness measurements were significantly higher in patients with MASLD (both *p* ≤ 0.001) (Table 2).

**Table 2:** Factors associated with MASLD among T2DM patients.

Variables	MASLD group (*N* = 103), *n* (%)	Group without MASLD (*N* = 46), *n* (%)	*p*
**Age, mean ±SD**	56.5 ± 10.9	61.4 ± 13.3	**0.019**
Sex			0.254
Female	70 (73.7)	25 (26.3)	
Male	33 (61.1)	21 (38.9)	
Lifestyle habits			
Smoking	16 (15.2)	9 (20.5)	0.437
Alcohol consumption	01 (01.0)	0 (00.0)	1.000
**Insulin**	46 (43.8)	18 (40.9)	0.744
**Oral antidiabetic drugs**	90 (85.7)	38 (86.4)	0.917
Metformin	90 (85.7)	35 (79.5)	0.350
Sulfonamides	28 (26.7)	14 (31.8)	0.524
Other oral antidiabetic agents	11 (10.5)	06 (13.6)	0.580
**Degenerative complications**	41 (39.0)	17 (38.6)	0.963
• Retinopathy	26 (24.8)	06 (13.6)	0.131
• Nephropathy	16 (15.2)	05 (11.4)	0.535
• Neuropathy	24 (22.9)	10 (22.7)	0.986
• Stroke	03 (02.9)	01 (02.3)	1.000
• Peripheral arterial disease of the lower limbs	06 (05.8)	01 (02.3)	0.674
• Acute coronary syndrome	11 (10.5)	02 (4.5)	0.394
**BMI (kg/m**^**2**^**), mean ±SD** (n = 144)	31.9 ± 5.1	27.3 ± 4.5	**0.001**
**Obesity**	63 (61.8)	11 (26.2)	**0.001**
**Abdominal obesity** (*n* = 143)	86 (86.0)	32 (74.4)	0.095
**Waist circumference (cm), median [IQR]**	100 [100–110]	98 [88–100]	**0.001**
Female	100 [97–110]	98 [90–100]	**0.012**
Male	99 [90–110]	92 [85–100]	0.092
**Systolic blood pressure (SBP)(mmHg), median [IQR]**	130 [120–140]	130 [120–140]	0.532
**Diastolic blood pressure (DBP)(mmHg), median [IQR]**	70 [70–80]	70 [65–80]	0.143
**White blood cell count /mm**^**3**^ **(WBC), median [IQR]**	7100 [5600–8300]	7350 [5500–8950]	0.723
**Hemoglobin (Hb)(g/dL), median [IQR]**	13 [12–13.9]	12.8 [11.9–13.5]	0.198
**Platelet count/μL (PLT), median [IQR]**	244000 [163750–260750]	212750 [177000–300000]	0.170
**Platelet distribution width (PDW)(%), median [IQR]**	9.4 [8.9–10.0]	9.2 [8.9–10.3]	0.798
**Fasting blood glucose(mmol/L), median [IQR]**	10.5 [8.3–14.2]	9.4 [7.5–13.3]	0.419
**Glycosylated hemoglobin(HbA1c)(%), median [IQR]**	9.2 [8–10.7]	9.5 [7.9–11.3]	0.629
**Triglycerides(mmol/L), median [IQR]**	1.6 [1.2–2]	1.2 [0.9–1.6]	**0.001**
**Total cholesterol(mmol/L), median [IQR]**	4.5 [3.8–5.5]	4.1 [3.3–4.7]	**0.001**
**High density lipoprotein (HDL) cholesterol(mmol/L), median [IQR]**	1.1 [1–1.3]	1.1 [0.9–1.4]	0.735
**Aspartate aminotransferase (AST)(UI/L), median [IQR]**	20 [15–26]	18.5 [14–23.7]	0.202
**Alanin aminotransferase (ALT)(UI/L), median [IQR]**	18.5 [13–30]	15 [11.2–22]	**0.018**
**Gamma–glutamyl transferase(GGT)(UI/L), median [IQR]**	26 [18–35]	18 [14–30.7]	**0.006**
**Alkaline phosphatase (ALP)(UI/L), median [IQR]**	74 [63–91.7]	76.5 [58.2–90.2]	0.952
**Total bilirubin(UI/L), median [IQR]**	9.5 [7–12]	10 [7–12]	0.878
**Prothrombin time(%), median [IQR]**	98 [91.5–100]	100 [94–100]	0.245
**Albumin(g/L), median [IQR]**	41.5 [39.2–45]	42 [40–43]	0.968
**Ferritin(μg/ L), median [IQR]**	53 [31.2–80.5]	62 [31.5–82.5]	0.783
**Controlled Attenuation Parameter (CAP)(dB/m)**, **mean ±SD**	297.9 ± 35.8	203 ± 33.3	**0.001**
**Liver stiffness measurement (LSM)(kPa), median [IQR]**	5.6 [4.1–6.9]	4.1 [3.5–5.4]	**0.001**
**Significant fibrosis**	26 (25.2)	6 (13.0)	0.131

Bold values indicate statistical significance (*p* < 0.05).

**Figure 1:**
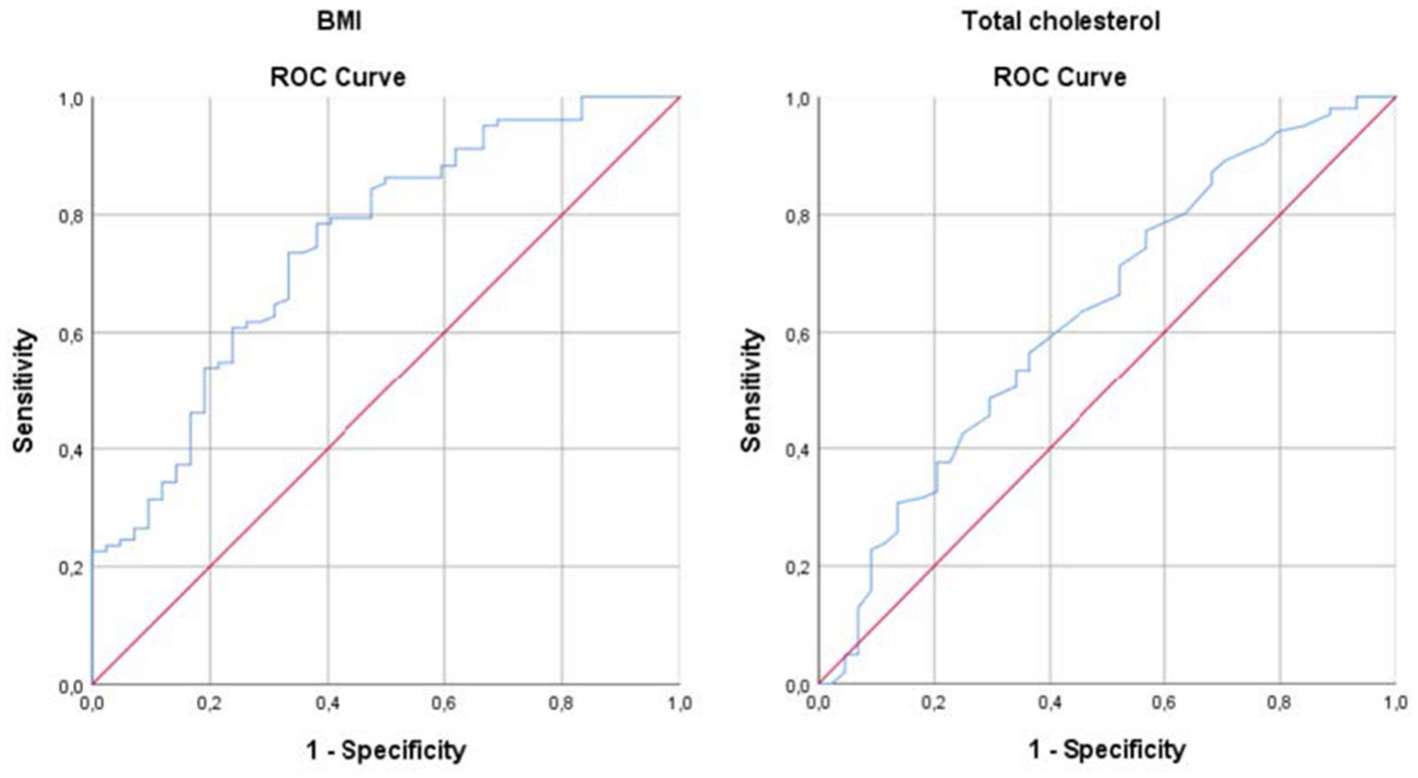
ROC-based BMI and cholesterol cut-offs in type 2 diabetes patients with MASLD.

Multivariable analysis identified BMI [aOR = 1.25; 95% CI (1.13–1.38); *p* < 0.001] and total cholesterol [aOR = 1.69; 95% CI (1.15–2.48); *p* = 0.007] as independent predictors of MASLD, with significantly increased odds associated with higher values of both parameters (Table 3). ROC analysis identified body mass index ≥27.8 kg/m^2^ and total cholesterol ≥3.95 mmol/L as predictors of hepatic steatosis. The BMI threshold had a sensitivity of 78.4%, specificity of 62.0%, and an area under ROC (AUROC) of 0.748 [95% CI (0.660–0.836); *p* < 0.001], whereas the cholesterol threshold showed a sensitivity of 71.3%, specificity of 50.0%, and an AUROC of 0.635 [95% CI (0.534–0.735); *p* = 0.010] (Figure 1).

**Table 3:** Univariate and multivariate logistic regression analysis of factors associated with MASLD.

Variable	Univariate analysis (*p*–value)	Multivariate analysis: aOR (95% CI)	*p*–value	VIF
Age	0.019	0.970 (0.930–1.011)		1.12
BMI	< 0.001	1.25 [1.13–1.38]	< 0.001	2.75
Triglycerides	0.001	2.507 (1.275–4.929)		1.12
Total cholesterol	0.001	1.69 [1.15–2.48]	0.007	1.3
ALT	0.018	1.028 (0.984–1.074)		1.78
GGT	0.006	0.991 (0.976–1.007)		1.67

### Liver stiffness and fibrosis risk stratification

The median liver stiffness measurement was 5.0 kPa (IQR 4.0–6.5). Applying the dual-cutoff strategy recommended by the EASL guidelines, advanced fibrosis was confidently ruled out (LSM < 8 kPa) in 82.6% (*n* = 123) of patients. Conversely, significant fibrosis could not be ruled out (LSM≥8 kPa) in 17.4% [95% CI (11.7–24.5), *n* = 26] of the sample. Among these, 13.4% (*n* = 20) fell into the intermediate-risk zone (LSM 8–11.99 kPa) where advanced fibrosis cannot be excluded, and 4% (*n* = 6) were classified as being at high risk of advanced fibrosis (LSM ≥ 12 kPa).

In univariate analysis, advanced fibrosis was associated with higher BMI (*p* = 0·007), increased waist circumference (*p* = 0·014), higher systolic blood pressure (*p* = 0·050), and higher platelet distribution width (*p* = 0·012). Patients with severe fibrosis had significantly higher AST, ALT, and GGT levels (all *p* < 0.001), as well as markedly higher liver stiffness measurements (*p* < 0.001) (Table 4).

**Table 4:** Factors associated with advanced fibrosis (MASLD at risk) among T2DM patients.

Variables	Advanced fibrosis group (LSM ≥ 8kPa)	Group without advanced fibrosis (LSM < 8Kpa)	P
**Age, mean ±SD**	56.6 ± 14.1	58.3 ± 11.2	0.489
Sex			0.281
Female	23(24.2)	72(75.8)	
Male	9(16.7)	45(83.3)	
Lifestyle habits			
Smoking	3(9.4)	22(18.8)	0.206
Alcohol consumption	00(00.0)	01(0.9)	1.000
**Insulin**	17(53.1)	47(40.2)	0.190
**Oral antidiabetic drugs**	25 (78.1)	103 (88.0)	0.254
Metformin	24 (75.0)	101 (86.3)	0.123
Sulfonamides	3 (9.4)	39 (33.3)	**0.008**
Other oral antidiabetic agents	2 (6.3)	15 (12.8)	0.470
**Degenerative complications**	13 (40.6)	45 (38.5)	0.824
• Nephropathy	9 (28.1)	23 (19.7)	0.301
• Neuropathy	5 (15.6)	16 (13.7)	1.000
• Stroke	7 (21.9)	27 (23.1)	0.886
• Peripheral arterial disease of the lower limbs	0 (00.0)	4 (3.4)	0.578
	0 (00.0)	7 (6.0)	0.346
• Acute coronary syndrome	4 (12.5)	9 (78.5)	0.478
**BMI(kg/m**^**2**^**), mean ±SD** (*n* = 144)	32.9 ± 5.5	30.0 ± 5.2	**0.007**
**Obesity**	19 (63.3)	55 (48.2)	0.141
**Obesity and overweight**	28 (93.3)	97 (85.1)	0.377
**Abdominal obesity (*****n*** **= 143)**	28 (93.3)	90 (85.1)	0.079
**Waist circumference (cm), median [IQR]**	102.5 [98–110]	98 [90–105]	**0.014**
**Female**	102.5 [98–110]	99 [94–105]	0.068
**Male**	101.5 [98–110.2]	95 [88–104.5]	0.127
**Systolic blood pressure (SBP) (mmHg), median [IQR]**	132 [130–150]	130 [120–140]	**0.050**
**Diastolic blood pressure (DBP) (mmHg), median [IQR]**	70 [70–80]	70 [65–80]	0.110
**White blood cell count/mm**^**3**^ **(WBC), median [IQR]**	6700 [4950–8100]	7300 [5575–8800]	0.206
**Hemoglobin (Hb) (g/dL), median [IQR]**	13 [11.9–13.9]	12.7 [12.1–12.7]	0.855
**Platelet count/μL (PLT), median [IQR]**	193000 [114500–193000]	243500 [179250–290000]	0.085
**Platelet distribution width (PDW)(%), median [IQR]**	9.7 [9.3–11.1]	9.2 [8.8–10]	**0.012**
**Fasting blood glucose (mmol/L), median [IQR]**	12.1 [7.9–14.2]	10.2 [8.3–13.8]	0.688
**Glycosylated hemoglobin (HbA1c) (%), median [IQR]**	8.95 [7.1–10.7]	9.4 [8.1–11.2]	0.319
**Triglycerides (mmol/L), median [IQR]**	1.2 [0.9–1.6]	1.5 [1.1–2]	0.061
**Total cholesterol (mmol/L), median [IQR]**	4.2 [3.3–5.2]	4.4 [3.7–5.4]	0.148
**High density lipoprotein (HDL) cholesterol (mmol/L), median [IQR]**	1 [0.9–1.3]	1.1 [1–1.4]	0.219
**Aspartate aminotransferase (AST) (UI/L), median [IQR]**	28 [22–50]	17 [14–22]	**0.001**
**Alanin aminotransferase (ALT) (UI/L), median [IQR]**	31 [22–55]	16 [12–22.5]	**0.001**
**Gamma-glutamyl transferase(GGT)(UI/L), median [IQR]**	39.5[27-62]	20[16-30]	**0.001**
**Alkaline phosphatase (ALP) (UI/L), median [IQR]**	76[67-102]	74[60-89.2]	0.291
**Total bilirubin(UI/L), median [IQR]**	10.5[8-15.2]	9[7-12]	0.072
**Prothrombin time(%), median [IQR]**	97[91-100]	100[95-100]	0.075
**Albumin(g/L), median [IQR]**	42[40-45]	41[39-44]	0.457
**Ferritin(μg/ L), median [IQR]**	53[31.2-69.2]	56[31.5-86.5]	0.708
**Controlled Attenuation Parameter (CAP) (dB/m), mean ±SD**	284.9 ± 54.7	267.5 ± 55.1	0.115
**Liver stiffness measurement (LSM)(kPa), median [IQR]**	9.4[8.1-11.7]	4.4[3.8-5.6]	**0.001**
**Steatosis**	26(81.3)	79(67.5)	0.131

Bold values indicate statistical significance (*p* < 0.05).

**Figure 2:**
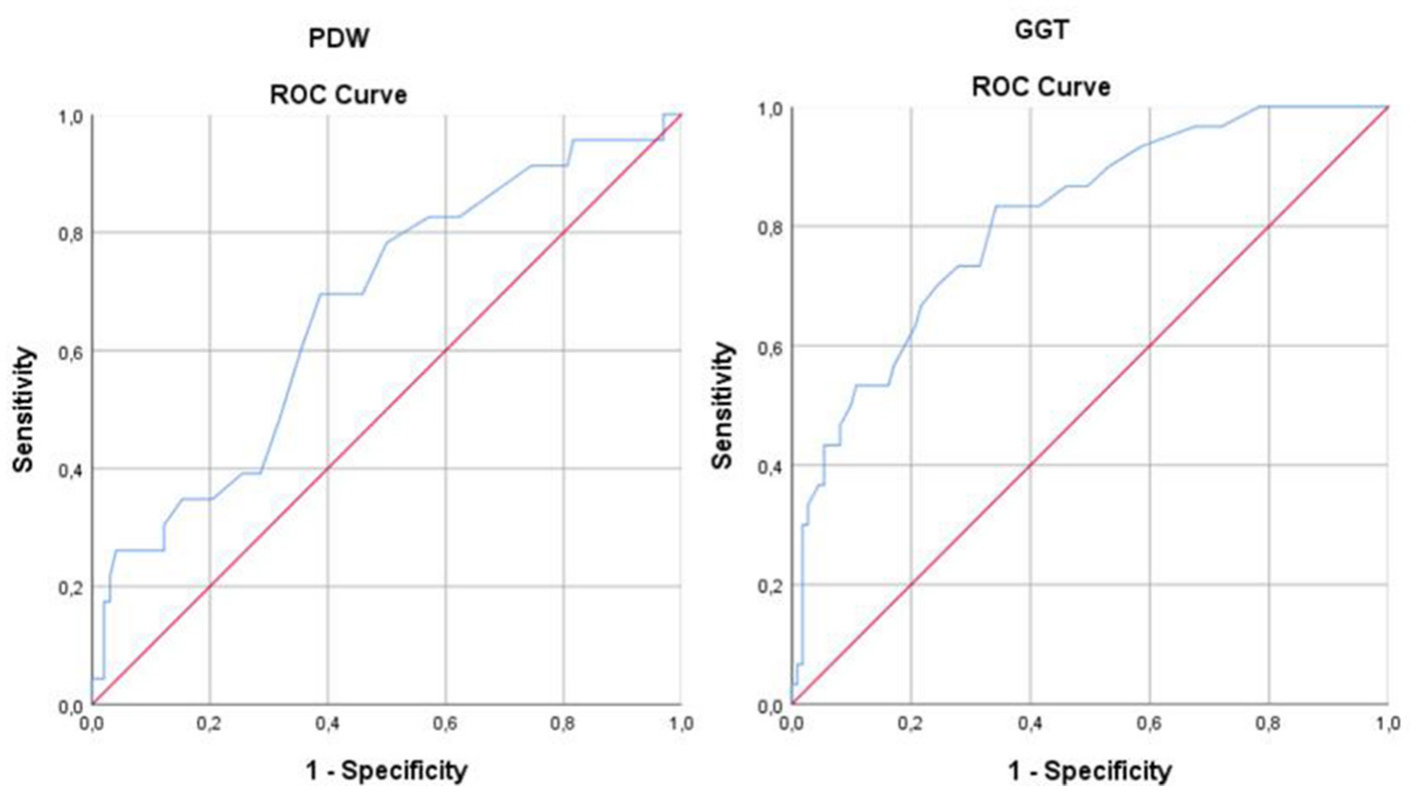
ROC-derived PDW and GGT thresholds in type 2 diabetes patients with significant hepatic fibro.

In multivariable analysis, platelet distribution width (PDW) and GGT were independently associated with severe hepatic fibrosis. Higher PDW was associated with nearly a threefold increase in the odds of severe fibrosis (aOR = 2.78; 95% CI: 1.08–7.21; *p* = 0.035), while increasing GGT levels were also significantly associated with severe fibrosis (aOR = 1.12; 95% CI: 1.03–1.21; *p* = 0.006) (Table 5). ROC curve analysis identified PDW and GGT thresholds of 9.5% and 25.5 UI/L, respectively, as predictors of hepatic fibrosis. The PDW threshold showed a sensitivity of 70%, specificity of 61.2%, and an AUROC of 0.669 [95% CI (0.547–0.791); *p* = 0.010], whereas the GGT threshold had a sensitivity of 83.3%, specificity of 65.8%, and an AUROC of 0.810 [95% CI (0.547–0.791); *p* < 0.001] (Figure 2).

**Table 5:** Univariate and multivariate logistic regression analysis of factors associated with of significant liver fibrosis in patients with T2D.

Variable	Univariate analysis (*p*–value)	Multivariate analysis: aOR (95% CI)	*p*–value	VIF
Sulfonamides	0.008			1.00
BMI	0.007			3.98
Waist circumference	0.014			3.56
Systolic blood pressure (SBP)	0.050			2.11
Platelet distribution width (PDW)	0.012	2.78 [1.08–7.21]	0.035	2.77
Aspartate aminotransferase	0.001			2.27
Alanin aminotransferase	0.001			2.22
Gamma–glutamyl transferase (GGT)	0.001	1.12 [1.03–1.21]	0.006	1.55

## Discussion

In our study, we observed a MASLD prevalence of 69.1%, and advanced fibrosis could not be ruled out (LSM ≥ 8 kPa) in 17.4% of patients with T2D. These findings underscore the critical need for systematic screening in this high-risk population and identify readily available clinical and biological parameters associated with advanced liver disease.

The demographic and metabolic profile of our study characterized by a mean age of 57.9 years, a female predominance (63.8%), and a high prevalence of obesity (51.4%), abdominal obesity (82.5%), and arterial hypertension (59.1%) is consistent with the typical clustering of cardiometabolic risk factors in T2D populations.

The suboptimal glycemic control, reflected by a median HbA1c of 9.4%, suggests a longstanding or poorly managed diabetes, providing a critical context for interpreting the high rates of hepatic complications.

We observed a MASLD prevalence of 69.1% based on CAP criteria. This prevalence aligns with the upper range of global estimates, which suggest that 55–70% of individuals with T2D have MASLD. (4, 7)

The severity of hepatic steatosis was notable, with over 40% (44.3%) of patients exhibiting grade 3 (S3) steatosis. This reinforces the concept that T2D is not merely a risk factor for MASLD but is associated with more severe hepatic phenotypes. This association is likely driven by the synergistic effects of hyperinsulinemia, insulin resistance, and hyperglycemia on hepatic *de novo* lipogenesis (17). Supporting this, Sasunova et al. demonstrated a close association between carbohydrate metabolism disorders and the progression of both steatosis and fibrosis, noting that while the severity of steatosis was similar between patients with prediabetes and T2D, advanced liver fibrosis was most frequent in the diabetic group (18).

Univariate analysis confirmed expected associations between MASLD and features of the metabolic syndrome, including higher BMI, waist circumference, triglycerides, total cholesterol, and liver enzymes (ALT, GGT). The association with younger age (*p* = 0.019) is an interesting finding that has been reported elsewhere (19), although it contrasts with a recent Italian study by Corrao et al. which found MASLD to be more prevalent in older males and did not identify age as a significant independent predictor (20). These discordances likely reflect differences in population characteristics, such as age distribution, sex ratios, and obesity patterns.

In multivariable analysis, BMI (aOR = 1.25; *p* < 0.001) and total cholesterol (aOR = 1.69; *p* = 0.007) emerged as the sole independent predictors of MASLD. The dominant role of BMI is expected, as obesity, particularly visceral adiposity, is a primary driver of insulin resistance and a major source of free fatty acids delivered to the liver (21). This finding is consistent with the 2025 NHANES analysis, which reported a significant positive association between the visceral adiposity index and MASLD risk (22). The independent predictive value of total cholesterol is particularly noteworthy. While hypertriglyceridemia is a more classic feature of insulin resistance-related dyslipidemia, our finding suggests that the overall cholesterol burden is a significant and independent contributor to hepatic steatosis in this T2D cohort. This may reflect an accumulation of cholesterol in hepatocytes, which can exacerbate lipotoxicity and steatosis (23, 24).

The ROC-derived BMI threshold of ≥27.8 kg/m^2^, which falls within the overweight range, was identified as a robust predictor of hepatic steatosis (AUROC = 0.748). This observation suggests that even modest elevations in BMI among patients with T2D warrant careful evaluation for MASLD. Additionally, a total cholesterol threshold of ≥3.95 mmol/L exhibited moderate specificity but reasonable sensitivity, underscoring its potential utility as a readily accessible clinical marker for identifying individuals at increased risk.

The proportion of patients in whom advanced fibrosis could not be ruled out was 17.4%, including 4% at high risk of advanced fibrosis (LSM ≥ 12 kPa). These findings confirm that patients with T2D constitute a high-risk population for advanced liver disease and aligns with other large cohort studies using vibration-controlled transient elastography. The inability to rule out advanced fibrosis represents a key predictor of long-term liver-related morbidity and mortality in patients with MASLD (25). Univariate analysis for advanced fibrosis risk showed associations with markers of obesity (BMI, waist circumference), liver injury (AST, ALT), and notably, with higher systolic blood pressure and PDW. These findings are consistent with a large multicenter study conducted in China, where advanced liver disease risk was much more prevalent in obese individuals with T2D (26).

In the multivariable model, two distinct factors were independently associated with the risk of advanced fibrosis. First, GGT (aOR = 1.12; *p* = 0.006) emerged as a strong independent predictor, with a high AUROC of 0.810 and good sensitivity (83.3%) at a threshold of 25.5 UI/L. GGT is a long-recognized marker of hepatobiliary disease and oxidative stress. Its strong association here reinforces its utility as a simple, low-cost biomarker for fibrotic progression in T2D, likely reflecting not only liver injury but also systemic oxidative stress, a key driver of fibrogenesis (27, 28). This is further supported by a Chinese study including 565 T2D patients with MASLD, where higher GGT levels were independently associated with advanced liver fibrosis and positively correlated with the FIB-4 index (29). Second, PDW (aOR = 2.78; *p* = 0.035) was associated with a nearly threefold increase in the odds of advanced fibrosis. PDW reflects variability in platelet size and is an indicator of platelet activation. While not a standard marker in most MASLD fibrosis scores (which typically use platelet count), emerging evidence suggests PDW may be a useful, readily available surrogate. As liver fibrosis progresses and portal hypertension develops, changes in the splanchnic circulation and thrombopoietin production can lead to alterations in platelet turnover and morphology, resulting in increased PDW (30). This finding suggests that PDW could be further explored as a component of novel biomarker panels for fibrosis. Although PDW was independently associated with significant fibrosis in our multivariate analysis, this finding should be considered hypothesis-generating. Further large-scale prospective studies are needed to validate PDW as a surrogate marker of liver fibrosis or portal hypertension before clinical application.

## Conclusion

This cross-sectional study brings to light the heavy and often silent burden of liver disease carried by patients living with T2D We found that 69.1% of participants met the criteria for MASLD, and advanced fibrosis could not be ruled out in 17.4% of patients, with 4% being at high risk (≥12 kPa). These findings underline the urgent call from international guidelines to implement systematic screening, confirming that patients with T2D face a markedly higher risk of progressing to advanced liver disease.

Diving deeper into the data, we uncovered that simple, everyday clinical markers (BMI and total cholesterol) stand as independent predictors of MASLD, with optimal thresholds set at 27.8 kg/m^2^ and 3.95 mmol/L, respectively. For significant fibrosis, GGT and PDW emerged as independent risk factors, with GGT proving to be a particularly reliable sentinel (AUROC 0.810).

## Data Availability

The original contributions presented in the study are included in the article/supplementary material, further inquiries can be directed to the corresponding author.
